# Effects of an Infectious Fungus, *Batrachochytrium dendrobatidis*, on Amphibian Predator-Prey Interactions

**DOI:** 10.1371/journal.pone.0016675

**Published:** 2011-02-02

**Authors:** Barbara A. Han, Catherine L. Searle, Andrew R. Blaustein

**Affiliations:** 1 Odum School of Ecology, University of Georgia, Athens, Georgia, United States of America; 2 Department of Zoology, Oregon State University, Corvallis, Oregon, United States of America; Institute of Marine Research, Norway

## Abstract

The effects of parasites and pathogens on host behaviors may be particularly important in predator-prey contexts, since few animal behaviors are more crucial for ensuring immediate survival than the avoidance of lethal predators in nature. We examined the effects of an emerging fungal pathogen of amphibians, *Batrachochytrium dendrobatidis*, on anti-predator behaviors of tadpoles of four frog species. We also investigated whether amphibian predators consumed infected prey, and whether *B. dendrobatidis* caused differences in predation rates among prey in laboratory feeding trials. We found differences in anti-predator behaviors among larvae of four amphibian species, and show that infected tadpoles of one species (*Anaxyrus boreas*) were more active and sought refuge more frequently when exposed to predator chemical cues. Salamander predators consumed infected and uninfected tadpoles of three other prey species at similar rates in feeding trials, and predation risk among prey was unaffected by *B. dendrobatidis*. Collectively, our results show that even sub-lethal exposure to *B. dendrobatidis* can alter fundamental anti-predator behaviors in some amphibian prey species, and suggest the unexplored possibility that indiscriminate predation between infected and uninfected prey (i.e., non-selective predation) could increase the prevalence of this widely distributed pathogen in amphibian populations. Because one of the most prominent types of predators in many amphibian systems is salamanders, and because salamanders are susceptible to *B. dendrobatidis*, our work suggests the importance of considering host susceptibility and behavioral changes that could arise from infection in both predators and prey.

## Introduction

In ecological communities, few responses are more important for immediate prey survival than contending with lethal predators. In this context, infectious agents such as parasites and pathogens can influence host anti-predatory behaviors with important consequences for predator-prey interactions. For example, several studies reveal that infection may lead to behavioral or physiological changes that make prey more conspicuous and therefore more vulnerable to predation [1], [2], [3]. In examples where predators can acquire pathogens from prey, evidence exists that predators may decrease their risk of acquiring infection by avoiding infectious prey and/or selectively consuming non-diseased prey [4], [5], [6]. Furthermore, theoretical investigations corroborate empirical conclusions that infection can act through both prey and predator behaviors to influence infection prevalence and host population dynamics [7], [8], [9], [10].

A novel amphibian pathogen provides an ideal system to examine how a generalist infectious agent may impact predator-prey interactions. *Batrachochytrium dendrobatidis* (Bd) [11], [12] is a globally distributed fungal pathogen that infects a broad diversity of amphibian host species worldwide (lists of host species available at http://www.spatialepidemiology.net/bd/). Though associated with prominent declines and extinctions of some amphibian populations (reviewed in [13], [14]), infection is not invariably lethal. For example, while extirpation has occurred for multiple populations in the Sierra Nevada (*Rana muscosa*, [15]), and over 90% of stream-dwelling amphibian species disappeared as a result of Bd epizootics in Panama (e.g., [16]), populations of other host species persist with low infection prevalence and without symptoms of disease in other regions around the world (e.g., [17], [18], [19]). Even with low-level infection, there is mounting evidence that Bd alters a number of important host behaviors such as social aggregation, thermoregulation, and foraging activity in multiple species [20], [21], [22]. However, despite evidence from other predator-prey systems suggesting that infection can greatly impact both host population and infectious disease dynamics, the role of Bd in higher level community interactions, such as predator-prey interactions, has not been well-explored (but see [23], [24]).

Predator-prey interactions are often governed by a variety of chemical and visual cues [25], [26]. In aquatic environments, larval amphibians such as frog tadpoles can detect chemical cues that emanate directly from predators in addition to cues emitted from injured conspecifics (alarm cues). In combination with visual stimuli, these chemical signals provide prey with information about the risk of predation, facilitating appropriate behavioral responses [27], [28]. Cues from predators that depend primarily on vision for prey capture may cause tadpoles to seek refuge, whereas cues from vibration- or motion-detecting predators may cause tadpoles to drastically decrease activity levels [26]. The expression of anti-predator behaviors is threat-sensitive and context-dependent, but combinations of flight and refuge-seeking behaviors are common among many amphibian species [27], [28]. Accurate detection and adequate behavioral responses to the threat of predation are critical for prey that must balance the costs associated with hiding or inactivity. For amphibian larvae, engaging in anti-predator behaviors decreases the time spent foraging for food or actively thermoregulating to maximize developmental rates which ultimately translate to a smaller size at metamorphosis and decreased fitness [29].

To examine whether Bd infection affects amphibian predator-prey interactions, we measured two important anti-predator behaviors, activity rate and refuge use, in a series of controlled laboratory experiments testing the responses of larvae of four host species (frogs: *Anaxyrus boreas*, *Rana aurora*, *Rana cascadae*, *Pseudacris regilla*) to Bd infection. In many temperate aquatic habitats, the dominant predators of tadpoles are other amphibian species, which are also susceptible to Bd infection. In particular, salamanders are among the most important tadpole predators in many amphibian communities [30] and have been found infected with Bd in the wild [31], [32]. Since attacking and consuming infected prey could increase the risk of Bd transmission to these predators, salamanders might be expected to display different predation patterns based on the infection status of their tadpole prey. To examine this possibility we also conducted feeding trials to determine whether two salamander species (*Ambystoma macrodactylum* and *Taricha granulosa*) indeed consume potentially infectious prey; and to compare the rates of predation on infectious vs. uninfected tadpoles of *R. aurora*, *R. cascadae*, and *P. regilla*.

## Methods

### Tadpole hosts

Bd does not infect amphibian embryos, presumably because they lack keratin, a protein which is required by Bd for growth, reproduction, and survival [11]. To ensure infection-free animals, we collected partial clutches of several egg masses (>10) of *R. aurora* from permanent ponds in Monmouth, OR (Polk County, elevation 61 m; latitude/longitude: 44.84/-123.30). Several whole egg masses (>25) of *P. regilla* were collected from temporary ponds and wetlands in Corvallis, OR (Benton County, elevation 87 m; latitude/longitude: 44.56/-123.26). Partial clutches of several *R. cascadae* (>5) and *A. boreas* (>25) egg masses were collected from permanent water bodies in Deschutes County, OR (elevation 1951 m; latitude/longitude: 44.29/-121.55). Eggs were collected at early developmental stages 11–15 [33] and reared in 37 L aquaria containing filtered water with aeration. Upon hatching, conspecifics from separate clutches were mixed between multiple 37 L aquaria at densities of approximately 100 tadpoles per tank. Complete water changes were conducted approximately every 7 days. Tadpoles were fed a 2∶1 ratio of ground alfalfa pellets and fish flakes *ad libitum*. All animals were kept at 14–16°C on a 14∶10 hr light:dark photoperiod for the duration of the experiment. At developmental stages 28–29 [33], tadpoles were moved into several 11 L aquaria at densities of 20 tadpoles each for inoculation with either Bd or a control treatment.

### Inoculation regime

We used inoculation methods known to produce infection in larvae of these species [22]. Culture plates (1% Tryptone and agar) containing Bd (JEL strain 215) were flooded with 15 mL of filtered water for 20 minutes to allow the discharge of infectious zoospores from sporangia. The liquid contents from 2 flooded culture plates were added to each of six 11 L aquaria containing 20 tadpoles each. The culture plates were submerged for 1–2 seconds in water to ensure that zoospores were transferred from the culture plate to the aquarium. The procedure was repeated with control culture plates (1% Tryptone and agar without Bd) for six additional 11 L aquaria containing 20 animals each. Using a hemacytometer we counted an average concentration of 6.18×10^6^ zoospores/mL for three Bd culture plates. Tadpoles were exposed to treatments for 10 days and taken haphazardly from treatment aquaria for experiments. For brevity, we refer to tadpoles exposed to Bd as “Bd^+^” and tadpoles exposed to the control agar wash as “Bd^−^”. A subsample of Bd^+^ and Bd^−^ tadpoles used in activity rate and refuge use trials were analyzed using real-time quantitative PCR techniques based on [34]. DNA was extracted from excised mouthpart tissue from the following species (sample sizes analyzed from each treatment, Bd^+^ and Bd^−^): *R. aurora* (N = 5); *A. boreas* (N = 8); *P. regilla* (N = 6); *R. cascadae* (N = 8). We used a DNAeasy 96 well kit (Qiagen, Valencia, California) for DNA extraction, and quantified DNA concentration using a spectrophotometer (Nanodrop Technologies, Wilmington, Delaware). We used the ABI 7300 Real-time PCR system (Applied Biosystems, Foster City, California) for PCR reactions. Twenty five µL reactions contained: 5 µL of 20 ng/L template DNA and 20 µL of master mix (containing 900 nM forward and reverse primers, 125 nM MGB probe, and Taqman Master Mix). We obtained *Batrachochytrium* genome equivalent standards from D. Boyle [34] and included triplicates of each standard serially diluted on each plate (10^−1^, 10^0^, 10^1^, 10^2^) and a duplicate of the high standard (10^3^). Unknown samples were run in triplicate and values that differed by a coefficient of variation greater than 0.2 were rerun for greater accuracy. Values obtained from the real-time PCR reaction are mean BD zoospore genome equivalents (ge) per nanogram of excised mouthpart tissue. This measure accounts for differences in BD infection severity between species based on size alone (i.e., more mouthpart tissue containing more zoospores).

In feeding trial experiments, tadpole host tissues could not be retrospectively sampled to quantify infection severity, thus Bd^+^ and Bd^−^ designations refer only to exposure status in feeding trials.

### Predators

We chose predators that naturally co-occur in habitats from which eggs of the four host/prey species described above were collected. From low elevation ephemeral pools, we collected adult rough-skinned newts (*Taricha granulosa*; hereafter referred to as “*Taricha*”; mean snout-vent length of adults in this population: 69.4±1.4 mm (Biga et al, *unpublished*); Benton County, OR; elevation 87 m; latitude/longitude: 44.56/-123.26) as predators of *R. aurora* and *P. regilla* [35]. From permanent water bodies at higher elevations, we collected larval long-toed salamanders (*Ambystoma macrodactylum*; hereafter referred to as “*Ambystoma*”), a common predator of *R. cascadae* [36] and *A. boreas* tadpoles (pers. obs.). The mean snout-vent length of larvae used in this experiment was 37.2±0.41 mm. Larvae were collected from Deschutes County, OR (elevation 1951 m; latitude/longitude: 44.29/-121.55). *Taricha* were held in 37 L aquaria, two individuals per tank. Six *Ambystoma* larvae were housed in each of several 37 L aquaria separated by mesh compartments (12×20×25 cm) within aquaria to prevent cannibalism [37]. Since evidence is accumulating that many prey species can assess predation risk based on cues from predator diet [38], predators were fed a single species diet of tadpoles *ad libitum* for approximately one month prior to activity and refuge use experiments to accumulate species-specific chemical cues in the water. For example, one group of *Ambystoma* predators was fed a diet of only *R. cascadae* tadpoles for one month, while a separate group of *Ambystoma* was fed a strict diet of *B. boreas* for one month prior to trials on each of these prey species. Similarly, one group of *Taricha* predators was fed a strict diet of *P. regilla*, while another group was fed a strict diet of *R. aurora* for one month prior to trials on each of these prey species. Water containing predators contained a combination of predator cues and species-specific alarm cues from consumption of tadpoles of a single species, and we used water from these predator-holding tanks for our behavioral trials. We refer to this mixture of predator and tadpole alarm cues as “predator cue” hereafter. Neutral (control) cues were harvested from two 37 L aquaria containing approximately 28 grams of *Tubifex* worms in each tank (*Tubifex tubifex*) for all species except *R. aurora*. We chose *Tubifex* worms because they are not consumed by tadpoles, and are non-predatory detritivores that are common in many freshwater ecosystems [39]. The neutral cue for *R. aurora* trials was filtered water (as in [40]) because *Tubifex* worms were unavailable during trials for *R. aurora*. Predators were starved for 5 days prior to feeding trials to standardize hunger levels among individual predators.

### Activity rates and refuge use of tadpoles

To examine activity and refuge use, we filled opaque plastic chambers (31.5×11×20 cm) with 1 L filtered water. The chambers were placed atop grids visible through the bottom of the chambers (grid squares  = 25 mm^2^). Refuges were constructed using black polyvinyl chloride pipe cut into segments and fitted inside 20 mL glass beakers to form a dark tunnel. A single refuge was placed haphazardly in one corner of each chamber. Chambers containing 1 refuge and 1 tadpole from the appropriate treatment (Bd^+^ or Bd^−^, and predator cue (+/−) were left overnight to acclimate before commencing the experiment. Our experiment employed a 2×2 factorial design with four treatment combinations for each host species tested separately: Bd^+^ with predator cue, Bd^+^ with neutral cue, Bd^−^ with predator cue, Bd^−^ with neutral cue. Treatments were randomly assigned to chambers and replicated 30 times for each species except *R. aurora* (20 replicates for predator cue treatments, 25 replicates for neutral cue treatments). 500 mL of predator or neutral cue was added to chambers and left for 60 minutes prior to data collection. Prior to trials, we observed tadpoles of each species near, underneath, or inside of refuges (pers. obs.) ensuring that tadpoles were not repelled by the presence of constructed refuges and able to use refuges if they sensed a threat.

We recorded the number of gridlines crossed and the number of times each tadpole used refuge in 30-second intervals over 4 hours for a total of 8 observations per tadpole. To ensure independence of activity data from refuge use data, we counted the number of times the tadpole used refuge during the 30 second period and recorded the number of gridlines crossed by the tadpole when it was not using refuge. Black curtains surrounded test chambers to prevent observers from disrupting the animals. All trials were conducted between 1000 and 1400 hours. We used 2-way analyses of variance for statistical analyses of the mean activity rate and refuge use of tadpoles from Bd^+^ and Bd^−^ treatments for each species.

### Feeding trials

We tested whether two predators (*Ambystoma* and *Taricha*) consume infected prey, and whether predation rates differed between Bd^+^ and Bd^−^ prey for three species (*P. regilla*, *R. cascadae*, *R. aurora*). Feeding trials were not conducted on *A. boreas* because tadpoles and *Ambystoma* predators were not available in sufficient numbers during the timeline of the experiment. Rectangular plastic tubs (capacity: 39.7 L, size: 0.16×0.86×0.42 m) were filled with approximately 7.6 L filtered water and acclimated to cold-room temperature (14–16°C) for a minimum of 4 hours. 10 tadpoles from either Bd^+^ or Bd^−^ treatments were then added to each tub and allowed to acclimate to novel conditions overnight. At 0900 the next morning a single predator was added to each tub (*Ambystoma* for *R. cascadae* trials, and *Taricha* for *R. aurora* and *P. regilla* trials), and predation was allowed to occur without replacement of consumed prey. We recorded the number of tadpoles remaining in each tub every 60 minutes for a total of 240 minutes (4 observations). There were 7 replicates (predators) per treatment, and we used each predator only once.

We measured the number of tadpoles consumed by predators over time, and applied generalized linear model (GLM) analyses for each predator-prey combination, including both “time” and “predator ID” as sources of variation in our models. Models for each species identified a Poisson error structure and a log link function. The general model structure for all three predator-prey combinations was: *Number tadpoles consumed* ∼*Bd treatment* + *Time* + *ID* + (Bd treatment*Time).

Within feeding trials, the survival of each individual tadpole was not monitored over time. We therefore measured the time until at least half of the tadpoles (5 or more) had been consumed by the predator and fit Cox proportional hazard models to estimate how Bd affects the risk of at least half of the tadpoles being consumed by the predator. We also tested for differences between Bd^+^ and Bd^−^ survival curves for each prey species. All analyses were implemented in the *R* statistical computing environment, version 2.11.1 [41], and survival analyses used the *survival* package [42].

## Results

Quantitative PCR reactions indicate that inoculation methods produced infection in tadpoles exposed to Bd in our experiments, and ensured that controls were not infected with Bd. There was variation in infection severity between species, with *A. boreas* showing the most severe infections (0.53 ge), followed by *R. aurora* (0.35 ge), *P. regilla* (0.11 ge), and *R. cascadae* (0.01 ge). None of the Bd^−^ (control) tadpoles were infected with Bd.

**Figure 1:**
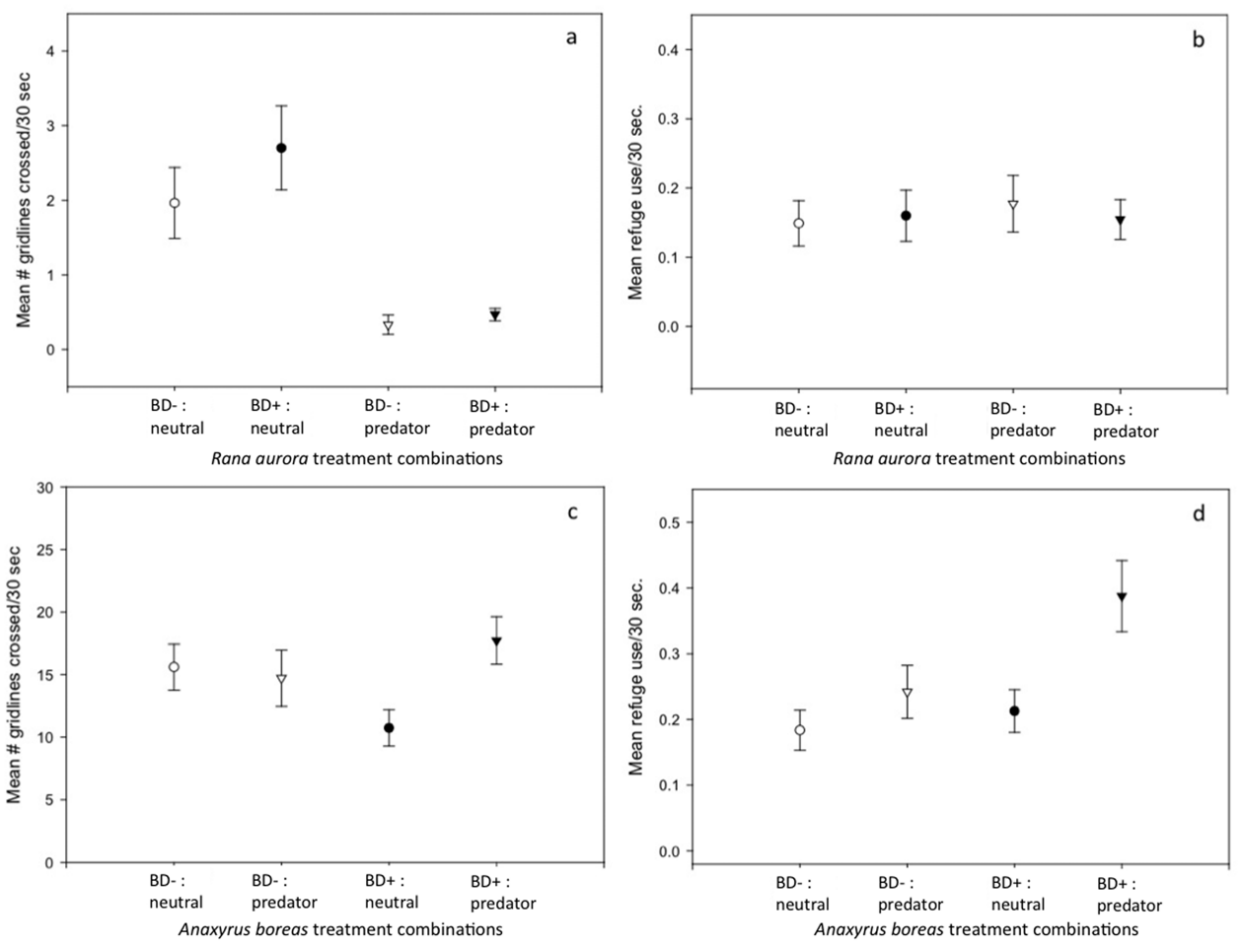
The mean number of gridlines crossed (a) and the mean number of times animals were observed using refuge (b) in 30 seconds for *Rana aurora* (a,b) and *Anaxyrus boreas* (c,d) in a 2×2 factorial design with two chemical cue treatments (predator, neutral), and two Bd treatments (Bd^+^, Bd^−^). *Rana aurora* tadpoles decreased activity in the presence of chemical cues from *Taricha* predators. Bd^+^ *Anaxyrus boreas* tadpoles were more active and used refuge more frequently when exposed to chemical cues of *Ambystoma* predators compared to neutral chemical cues.

Tadpole host behaviors in response to Bd and predator chemical cues varied across the four prey species tested. *Rana aurora* tadpoles exposed to predator cues were less active than those exposed to neutral cues. There was no effect of Bd on activity. There was also no effect of either Bd exposure or cue treatment on refuge use in *R. aurora* (Figure 1a,b). In *A. boreas* tadpoles there was a significant interaction between the Bd and cue treatments on activity rates, and significant main effects of Bd and cue treatments on refuge use (Table 1; Figure 1c,d). Tukey HSD post-hoc tests revealed that Bd^+^ *A. boreas* tadpoles exposed to predator cue were more active and used refuge more frequently compared to tadpoles in neutral cue treatments and compared to uninfected tadpoles. Neither activity rate nor refuge use differed between Bd or predator cue treatments for the remaining two species, *P. regilla* or *R. cascadae* (Table 1).

**Table 1:** Two-way ANOVA tables for activity (mean number of gridlines crossed/30 sec) and refuge use (the mean number of times animals were observed using refuge/30 sec) for larvae of four amphibian species in a 2×2 factorial design with two cue treatments (predator, neutral), and two exposure treatments to *Batrachochytrium* pathogen (Bd^+^ and Bd^−^).

		Activity				Refuge use			
Species	Source	Df	MS	F	*p*	Df	MS	F	*p*
*R. cascadae*	Bd	1	0.48	0.02	0.89	1	0.02	0.39	0.54
	Cue	1	0.12	0.01	0.94	1	0.19	3.27	0.07
	Bd*Cue	1	6.96	0.30	0.58	1	0.02	0.33	0.57
	Residuals	108	23.00			109	0.06		
*R. aurora*	Bd	1	4.16	0.99	0.32	1	0.00	0.01	0.91
	Cue	1	112.19	26.77	**<0.01**	1	0.00	0.10	0.75
	Bd*Cue	1	2.70	0.64	0.42	1	0.01	0.22	0.64
	Residuals	116	4.19			86	0.03		
*A. boreas*	Bd	1	25.48	0.24	0.63	1	0.23	4.71	**0.03**
	Cue	1	280.66	2.61	0.11	1	0.41	8.37	**<0.01**
	Bd*Cue	1	464.62	4.32	**0.04**	1	0.10	2.09	0.15
	Residuals	116	107.49			116	0.05		
*P. regilla*	Bd	1	8.04	0.96	0.33	1	0.00	0.00	0.96
	Cue	1	13.93	1.67	0.20	1	0.02	0.27	0.61
	Bd*Cue	1	0.02	0.00	0.96	1	0.03	0.49	0.48
	Residuals	114	8.36			116	0.06		

**Figure 2:**
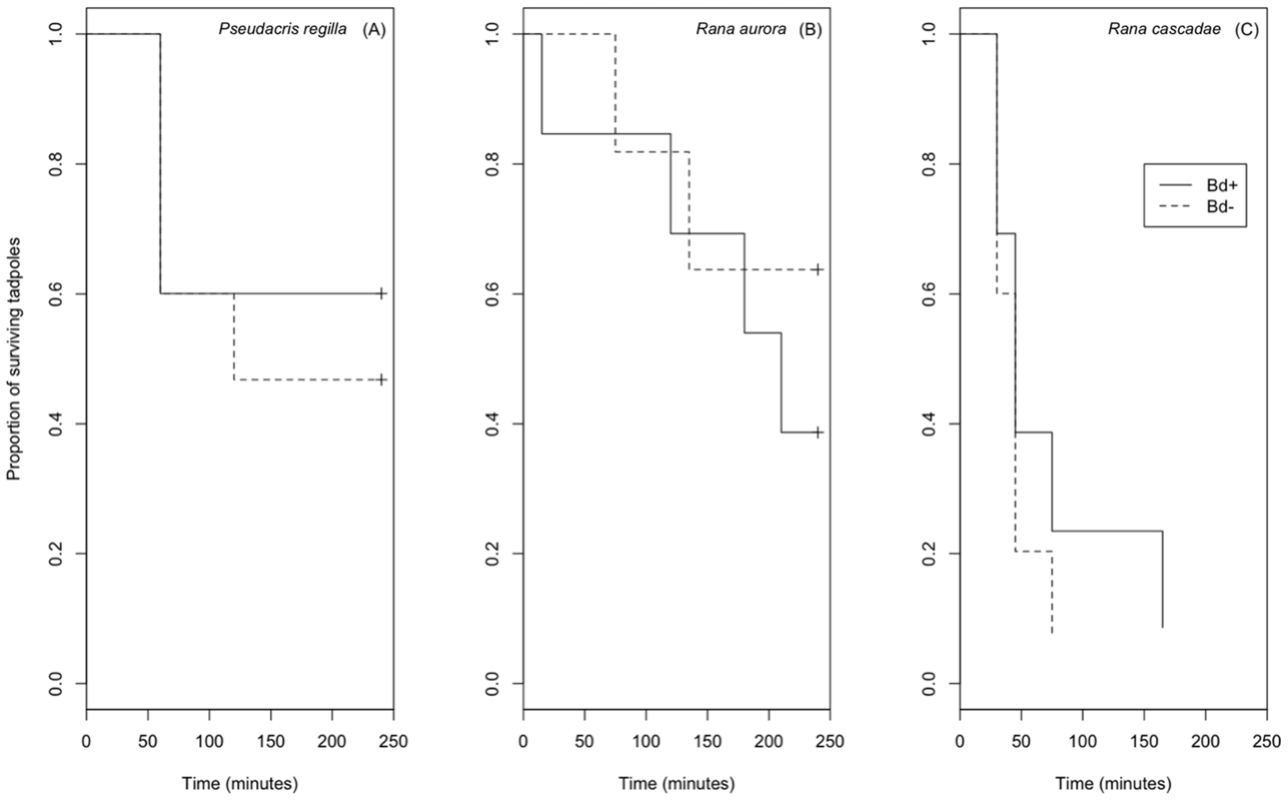
Survival of tadpole prey of three amphibian species (*P. regilla*, *R. aurora*, and *R. cascadae*) from two Bd treatments (Bd^+^, Bd^−^) during feeding trials with two predator species (*Taricha granulosa* and *Ambystoma macrodactylum*) in the following species combinations: *Taricha*-*P.regilla*; *Taricha*-*R. aurora*; *Ambystoma*-*R. cascadae*). The symbol (+) at the end of survival curves for *P. regilla* and *R. aurora* denotes censored data, where some prey survived beyond the end of the feeding trial. All *R. cascadae* tadpoles were consumed by *Ambystoma* predators during feeding trials.

In feeding trials, predators consumed tadpoles from both Bd^+^ and Bd^−^ treatments in all three prey species tested (*P. regilla*, *R. cascadae*, and *R. aurora*; Figure 2). More Bd^+^ *R. aurora* tadpoles were consumed by predators compared to controls (z = 2.94, p = 0.003), with variation between individual predators contributing to this effect (z = 2.53, p = 0.012). For *R. cascadae* tadpoles, there was a decrease in the total number of tadpoles consumed over time (z = −2.51, p = 0.012), but no effect of the Bd treatment. Predation of *P. regilla* was not explained by Bd treatment, Time, or variation between individual predators. Also, Bd*Time interactions were not significant for any of the three predator-prey combinations tested, indicating that predation rates were not influenced by Bd status of the prey. A two-sample power analysis shows the minimum detectable effect size (i.e., the estimated magnitude of difference between Bd^+^ vs. Bd^−^ prey consumed) is 0.44 given our sample sizes for each predator-prey combination (power = 0.8, significance level = 0.05). This retrospective power analysis shows that our design was sufficient to detect ‘medium’ effects of predators on prey populations (*sensu* [43]), but suggests that larger sample sizes may increase the ability to detect smaller effect sizes.

Prey survival curves did not differ significantly between Bd treatments for any prey species (*R. aurora*, X^2^ = 0.5, df = 1, p = 0.498; *R. cascadae*, X^2^ = 0.8, df = 1, p = 0.365; *P. regilla*, X^2^ = 0.2, df = 1, p = 0.644). Cox proportional hazards tests showed that Bd increased predation risk in *R. aurora* (by 1.8 times ±2.4), and decreased predation risk in *P. regilla* (by 0.75 times ±2.1) and *R. cascadae* (by 0.6 times ±1.8) compared to the control treatments, but these differences were not statistically significant (Table 2).

**Table 2:** Survival analysis using Cox proportional hazards of amphibian prey species from predation by two salamander species: *Taricha granulosa* predators on tadpoles of *Pseudacris regilla* and *Rana aurora*, and *Ambystoma macrodactylum* predators of *Rana cascadae* tadpoles.

Prey species	coef	exp(coef)	SE(coef)	*z*	*p* value
*P. regilla*	−0.28	0.75	0.76	−0.377	0.71
*R. cascadae*	−0.39	0.68	0.59	−0.662	0.51
*R. aurora*	0.58	1.79	0.87	0.669	0.50

## Discussion

It is clear that the interactions between parasites, predators, and prey in an ecological community are complex, and may be an important driver of infection and population dynamics in many systems [7], [44]. As a first investigation of the potential impacts of a widespread infectious fungus on predator-prey behavioral interactions in amphibian communities, we examined how two common anti-predator behaviors in four species of prey were affected by predator chemical cues and exposure to Bd. We also examined whether predators respond to infection risk by consuming uninfected prey more frequently compared to infected prey. Our results showed clear interspecific differences in the anti-predator behaviors of amphibian larvae in response to chemical cues of predators. We found that Bd exposure changed anti-predator behaviors in the tadpoles of one species, *A. boreas*, but not in the other three species we examined (*P.regilla, R. aurora*, and *R. cascadae*). Feeding trials revealed that salamander predators (*Taricha* and *Ambystoma*) consumed both Bd^+^ and Bd^−^ tadpole prey of these latter three species at similar rates, and that predation risk among prey was not influenced by infection status.

*Anaxyrus boreas* tadpoles in Bd^+^ treatments reacted strongly to predator cues by increasing their activity rates and refuge use. A number of studies have demonstrated that *A. boreas* is particularly susceptible to Bd infection at both larval and post-metamorphic life stages (e.g., [45], [46]), and corroborate our observations of hyperactivity among infected tadpoles [22]. Although we were unable to formally quantify this behavior, we note that hyperactivity may more accurately be described as erratic and unpredictable swimming behavior characterized by darting quickly around the test chamber and visiting refugia seemingly randomly during these bouts of erratic activity. We speculate that hyperactivity/erratic activity of Bd^+^ *A. boreas* tadpoles exposed to predator cues may arise through a synergism between incidental physiological effects of Bd infection and a behavioral response to predation risk. Tadpoles with increased activity rates that are also frequenting available refugia can decrease predation risk by making capture mechanically more difficult for predators [47], [48]. Moreover, large aggregations of *A. boreas* that typically occur in nature ([e.g., 49]) can create a confusion effect that helps to evade predation (reviewed in [50], [51]), though we note that group behavior may differ from that of individuals observed in a laboratory setting. In contrast, Bd^−^ tadpoles exposed to predator chemical cues may reflect behaviors similar to the other two species we examined (*P. regilla*, *R. cascadae*), requiring more information before responding with potentially costly anti-predator behaviors (e.g., [52]).

Based on several previous studies (reviewed in [26]), we expected all four species to respond to predator chemical cues by decreasing activity rates and hiding more frequently. However, only two of the species we examined (*R. aurora* and *A. boreas*) fulfilled these expectations. The other two species (*P. regilla*, *R. cascadae*) showed no detectable differences in activity or refuge use in response to predator chemical cues. The anti-predator behaviors that we measured, reducing activity and utilizing refugia, present significant costs to larval amphibians by decreasing the amount of time tadpoles spend foraging and actively thermoregulating to maximize growth rates. In ephemeral habitats, long periods of inactivity or hiding can depress growth and developmental rates, ultimately decreasing fitness [53], [54]. To reduce the costliness of these anti-predator behaviors, prey assess predation risk through multiple sources of information, using both visual and chemical cues in the environment [55]. Thus, one possibility is that the latter two species required more information signaling predation threat to elicit any detectable changes in anti-predator behaviors (e.g., more concentrated and/or longer exposure to predator chemical cues, or the addition of visual stimuli [56], [57]).

Amphibian predators (including *Ambystoma* and *Taricha* species) are susceptible to Bd infection [31], [32], [58]. Thus, Bd transmission to salamander predators through consuming infected prey is a realistic possibility that may contribute to infection dynamics in amphibian communities. To our knowledge, this possibility remains unexplored. The results from feeding trials suggest that neither *Taricha* nor *Ambystoma* salamander species we tested avoid consuming infected tadpole prey. On the contrary, predators consumed tadpoles from both Bd^+^ and Bd^−^ treatments at statistically indistinguishable rates, and showed no differences in the total number of tadpoles consumed among the three prey species tested (*P. regilla*, *R. aurora*, *R. cascadae*). In addition, neither the rate nor the risk of predation differed across Bd treatments among the three predator-prey combinations we examined. These results suggest that selective predation (detectable through differences in predation rates on tadpoles among Bd treatments) may not be acting on this system, although it is important to note that the low infection levels found in *R. cascadae* may partially explain why *Ambystoma* predators are non-selective on this prey species. In contrast to a growing number of studies investigating the impacts of selective predation on infectious disease dynamics [e.g., 7,59], the ecological consequences of non-selective predation have not been examined widely. However, recent theoretical works suggest that even when predators do not select between hosts on the basis of infection status, equilibrium disease prevalence can increase in a population in a number of ecologically plausible scenarios, such as when infection is non-regulatory (i.e., when it does not influence population dynamics through host demography) [10]. Furthermore, selective predation may not allow for long-term persistence of prey populations that are also affected by infectious disease [4], implying the possibility that non-selective predation may be one mechanism contributing to the persistence of multiple host species and a shared infectious agent. We suggest that more detailed examinations of the patterns of selective vs. non-selective predation in natural communities may shed light on why some infected amphibian populations persist while others go extinct. Extending the patterns observed in our study to any persistent effects on prey populations and predator-prey interactions in natural systems will require closer examination of the degree of predator selectivity *per se* (e.g., through tests where predators make a distinct choice between consuming infected vs. uninfected prey); and the identification of additional infection-related drivers of prey population dynamics, such as how Bd infection affects predator as well as prey behaviors in semi-natural settings (e.g., capture efficiency of predators, and escape efficiency of prey).

Population declines of frogs and toads associated with Bd infection are precipitating important long-term changes to natural communities (e.g., ecosystem dynamics and community structure [60], [61]). Though Bd has often been considered a relatively superficial and non-lethal infection for larvae of most amphibian host species (but see [45]), it is clear that sub-lethal infection is sufficient to alter even basic behaviors such as schooling, thermoregulation [22], and foraging efficiency [20] in multiple species. Our study provides a timely first examination of how non-lethal Bd exposure can induce important behavioral changes contributing to predator-prey interactions in some host species (e.g., *Anaxyrus boreas*); and highlights an unexplored possibility that this pathogen may affect infection prevalence and community dynamics through insidious effects on amphibian predator-prey interactions.
